# Fibrosis and Src Signalling in Glaucoma: From Molecular Pathways to Therapeutic Prospects

**DOI:** 10.3390/ijms26031009

**Published:** 2025-01-24

**Authors:** Liam Bourke, Colm O’Brien

**Affiliations:** UCD Clinical Research Centre, Mater Misericordiae University Hospital, D07 R2WY Dublin, Ireland

**Keywords:** glaucoma, Src inhibition, fibrosis, extracellular matrix (ECM) remodelling, lamina cribrosa (LC), intraocular pressure (IOP), therapeutic targets

## Abstract

Glaucoma, a leading cause of irreversible blindness, is characterised by progressive optic nerve damage, with elevated intraocular pressure (IOP) and extracellular matrix (ECM) remodelling in the lamina cribrosa (LC) contributing to its pathophysiology. While current treatments focus on IOP reduction, they fail to address the underlying fibrotic changes that perpetuate neurodegeneration. The Src proto-oncogene, a non-receptor tyrosine kinase, has emerged as a key regulator of cellular processes, including fibroblast activation, ECM deposition, and metabolism, making it a promising target for glaucoma therapy. Beyond its well-established roles in cancer and fibrosis, Src influences pathways critical to trabecular meshwork function, aqueous humour outflow, and neurodegeneration. However, the complexity of Src signalling networks remains a challenge, necessitating further investigation into the role of Src in glaucoma pathogenesis. This paper explores the therapeutic potential of Src inhibition to mitigate fibrotic remodelling and elevated IOP in glaucoma, offering a novel approach to halting disease progression.

## 1. Introduction

Glaucoma, a primarily age-associated, progressive optic neuropathy, remains the leading cause of irreversible blindness worldwide [1]. With our increasingly aging global population, its prevalence is expected to increase significantly, from over 75 million cases in 2020 to an estimated 112 million by 2040, posing substantial socioeconomic challenges and underscoring the urgent need for novel therapeutic strategies [2]. Although recent advances have improved glaucoma treatments, current therapies universally focus on lowering elevated intraocular pressure (IOP), the principal risk factor for primary open-angle glaucoma (POAG). However, in many patients, IOP reduction alone is inadequate to prevent the progressive pathological remodelling of the extracellular matrix (ECM) within the lamina cribrosa (LC), ultimately resulting in continued vision loss. An alternative approach, which directly targets the ECM remodelling processes within the LC, offers a promising yet complex strategy [3]. Achieving this goal will require deeper insights into the molecular mechanisms driving LC remodelling, which we aim to investigate further in the future as a path to more effective therapeutic interventions. Elevated IOP and oxidative stress in glaucoma activate pro-inflammatory pathways, triggering cytokine release and promoting cellular damage and death [4,5]. While the exact mechanisms underlying glaucoma are complex and multifactorial, Src has been implicated in several processes that contribute to its pathophysiology. For example, Src regulates cytoskeletal dynamics, cell adhesion, proliferation, and survival, which are critical in maintaining trabecular meshwork (TM) function and ECM homeostasis [6]. Dysregulated Src activity may alter actin organisation, leading to changes in TM cell morphology, contraction, and adhesion, which might increase resistance in the conventional aqueous humour outflow pathway and elevate IOP [7]. Src activity is crucial in TGF-β-induced IOP elevation, and its inhibition may prevent Yes-associated protein (YAP)-driven fibrosis and modulate IOP by limiting its transcriptional activity [8,9]. Src has also been implicated in neurodegenerative processes in glaucoma, with its activation linked to retinal ganglion cell apoptosis—a key driver of optic nerve damage in the disease [10]. Targeting Src signalling presents a promising therapeutic approach for glaucoma treatment [11].

## 2. Src Proto-Oncogene Overview

The Src proto-oncogene, located on chromosome 20 (specifically 20q11.2 in humans), encodes a non-receptor tyrosine kinase [12,13]. First identified in the early 1970s, Src is one of the first oncogenes discovered and studied extensively in cellular transformation and cancer biology [14]. Eleven members of the human Src family tyrosine kinases (SFKs) have been identified, categorised into three groups [15]. Group I (SrcA), which includes Src, Fyn, Yes, and Fgr, and Group II (SrcB), which comprises Blk, Hck, Lck, and Lyn, are closely related. By contrast, Group III, consisting of Frk, Srm, and Brk, is more distantly related to the first two groups [16]. Src, Fyn, and Yes are ubiquitously expressed across all cell types, whereas Blk, Fgr, Hck, Lck, and Lyn are predominantly restricted to haematopoietic cells, and Srm is primarily found in keratinocytes. Frk is mainly expressed in bladder, brain, breast, colon, and lymphoid cells, while Brk is predominantly found in the colon, prostate, and small intestine, although it was originally isolated from a breast cancer cell line [17]. These proteins play critical roles in the signal transduction pathways that regulate apoptosis, cell cycle progression, cytoskeletal rearrangement, differentiation, development, the immune response, nervous system function, and transcription. Src and Src family kinases have been implicated in each of these processes [6,16].

Protein phosphorylation is the most widespread type of post-translational modification used in signal transduction [15]. Families of protein phosphatases catalyse the dephosphorylation of proteins, therefore making phosphorylation-dephosphorylation an overall reversible process [18]. Protein kinases play crucial regulatory roles in nearly every aspect of cell biology [15]. Src’s activation typically occurs through various pathways involving growth factors and cytokines [16,19].

**Figure 1:**
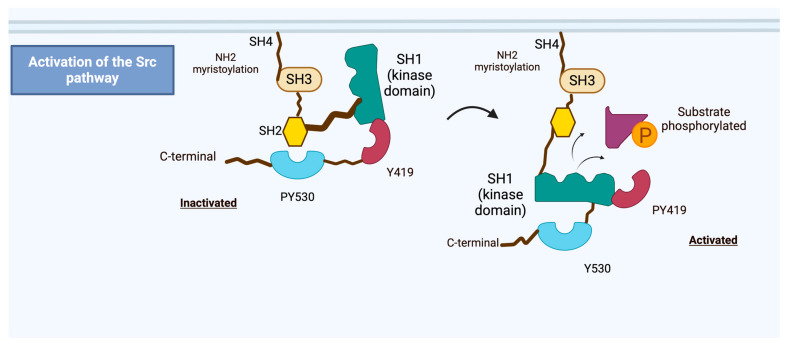
**Structure and activation of Src**. The Src protein has several key domains. The N-terminal SH4 domain contains a myristoylation sequence critical for membrane attachment. The SH3 domain interacts with proline-rich regions, facilitating protein–protein interactions, while the SH2 domain binds phosphorylated tyrosine residues, regulating Src activity and enabling interactions with other tyrosine-containing proteins. The SH2-linker region, situated between SH2 and SH1, further modulates Src activity by interacting with SH3. The SH1 domain contains the kinase domain, including the tyrosine residue Y419, the phosphorylation of which promotes Src activation (PY419). Src activity is tightly regulated by the phosphorylation state of the C-terminal tyrosine residue Y530. When Y530 is phosphorylated by C-terminal Src kinase (CSK), it binds to the SH2 domain, locking Src in an inactive conformation (PY530). Dephosphorylation of Y530 by protein tyrosine phosphatases induces open conformations of the SH1, SH2, and SH3 domains, leading to full activation of Src. Created in BioRender. Bourke, L. (2025) https://BioRender.com/i51t158 (accessed on 22 January 2025).

Structurally, Src contains several domains: the SH1 (catalytic) domain, the SH2 domain (which binds phosphotyrosine residues), the SH3 domain (which binds proline-rich sequences), and an N-terminal myristoylation site that targets it to the plasma membrane [6,16]. The principal phosphorylation sites of human Src consist of the activating phospho-Tyr419 (PYTyr419), which arises from autophosphorylation within the kinase domain by a neighbouring Src molecule, and the inhibitory phospho-Tyr530 (PYTyr530), located in the regulatory tail, which is phosphorylated by C-terminal Src kinase (Csk) or Csk homologous kinase (Chk) [20]. Dephosphorylation of this site leads to an open, active conformation, enabling its kinase activity and interaction with downstream effectors (Figure 1). Dephosphorylation can result from various factors, such as the action of protein tyrosine phosphatases or signalling cascades triggered by growth factors [21].

In fibroblasts, Src localises to endosomes, perinuclear membranes, secretory vesicles, and the cytoplasmic aspect of the plasma membrane, where it engages with a diverse array of growth factor receptors, integrins, and G-protein-coupled receptors [16]. Through these interactions, Src functions as a critical mediator in cellular signal transduction pathways [6,16]. The elevated expression of Src in platelets, which are anucleate cells, and in neurons, which are postmitotic, suggests that Src plays functional roles in cellular processes beyond those directly associated with cell division [16].

**Figure 2:**
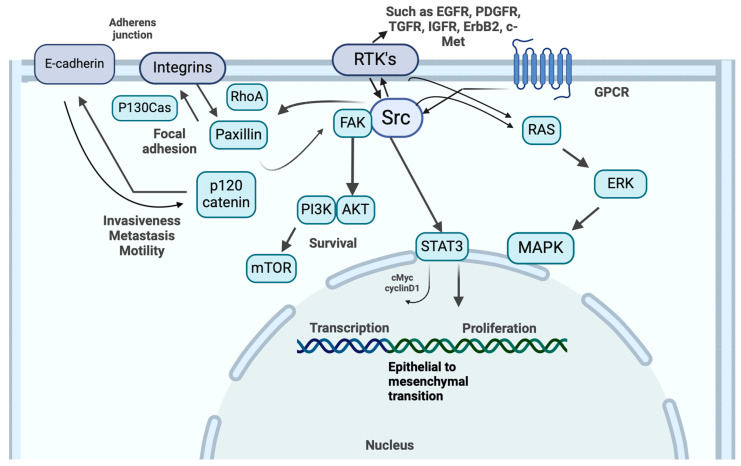
**Src intracellular signalling pathways**. Src kinases regulate diverse cellular processes, including adhesion, motility, proliferation, survival, and metastasis. Upon activation, Src stimulates key signalling pathways, such as FAK, PI3K-AKT, STAT3, RAS-ERK-MAPK, and integrins. In the trabecular meshwork, Src-mediated activation of these pathways contributes to fibrosis by promoting extracellular matrix (ECM) deposition, myofibroblast differentiation, and actin cytoskeletal remodelling. These changes impair conventional aqueous outflow, elevating intraocular pressure and exacerbating glaucoma pathophysiology. The figure highlights Src’s central role in coordinating these intracellular signalling networks and their influence on both normal cellular functions and pathological processes, such as carcinogenesis and fibrosis. Created in BioRender. Bourke, L. (2025) https://BioRender.com/y98l647 (accessed on 22 January 2025).

The association of Src with cancer is well established, particularly because of its roles in regulating cell growth, migration, and survival—all of which are fundamental processes in tumorigenesis [22,23] (Figure 2).

In normal cells, Src is under tight regulatory control, but in pathological conditions, this regulation is often lost, leading to constitutively active Src [24]. It can enhance entry into the cell cycle and indirectly promote the expression of cyclins that drive the progression from G1 to S phase [16,25]. Src has also been investigated as a key player in activating the ERK1/2 pathway in primary uveal melanoma (UM), suggesting that Src inhibition could be a viable therapeutic approach for primary UM [26]. However, the reduced reliance on Src and ERK1/2 signalling in metastatic UM cells indicated that Src inhibition may be less effective in treating metastatic disease, where alternative pathways drive proliferation.

In cancer, Src promotes various malignant phenotypes through multiple downstream pathways [23,27]. Src can bind to and phosphorylate the receptor tyrosine kinase (RTK) EGFR, leading to enhanced downstream oncogenic signals through pathways like PI3K/AKT and MAPK [28,29]. This Src-mediated EGFR activation contributes to the development of a more aggressive cancer phenotype in various tumour types [30]. RTK signalling pathways that are required for DNA synthesis and modulate receptor turnover, actin cytoskeleton dynamics, and cell motility are also regulated by Src [16]. In addition, Src can drive angiogenesis via vascular endothelial growth factor (VEGF) signalling, further supporting tumour growth [31].

Src not only plays an important role in mediating signalling from RTKs but also from G-protein-coupled receptors (GPCRs) [32]. For GPCR signalling, Src can directly associate with GPCRs or receptor-associated proteins, or they can be activated by GPCR-mediated transactivation of RTKs and focal adhesion complexes [32]. Src also play key roles in regulating cell adhesion and migration, in part through their interactions with integrins and molecules like focal adhesion kinase (FAK), paxillin, and p130Cas. Alterations in Src-mediated integrin signalling contribute to pathological cellular remodelling [32]. All of these processes contribute to pathological cellular migration. Dysregulated integrin signalling can propagate feedback loops that contribute to the establishment and progression of disease states, including glaucoma [33].

An integrated small interfering RNA (siRNA) screening approach identified nine kinases, including SGK1, as critical mediators of Src-induced cellular transformation [34]. In this context, Src has been shown to upregulate SGK1 expression, particularly in triple-negative breast cancer cells, which are characterised by a prominent Src family kinase-driven signalling network. Moreover, combined inhibition of Src and SGK1 significantly reduced colony formation and xenograft tumour growth, demonstrating enhanced efficacy compared to targeting either pathway in isolation [34]. These findings not only elucidate key mechanistic aspects of Src-driven pathogenesis but also offer valuable insights for the development of more effective therapeutic strategies, potentially applicable to other pathological conditions such as fibrosis or glaucoma.

Given its central role in cancer progression, Src is a promising therapeutic target, and several small-molecule inhibitors (such as saracatinib and dasatinib) have been developed to block its activity [35,36]. However, clinical trials have shown mixed results, indicating the complexity of targeting Src in cancer [27]. Concomitant targeting of the RAS/RAF/MEK/ERK pathway alongside PI3K/AKT/mTOR inhibitors may represent a promising therapeutic approach due to the substantial stimulatory crosstalk between these signalling cascades [37]. This is relevant to glaucoma, as many of the cellular and molecular mechanisms influenced by Src in cancer, such as cytoskeletal remodelling, mechanotransduction, and integrin-mediated adhesion, also play critical roles in glaucomatous pathology.

Src serves as a master regulator of glucose metabolism, coordinating metabolic adaptations to sustain diverse cellular processes and signalling pathways, similar to key molecules such as AKT, AMPK, mTOR, and HIF-1α [38]. Emerging evidence suggests that Src regulates the metabolism of biomolecules beyond glucose, including lipids and amino acids [39,40,41,42]. Src enhances metabolic flexibility by activating glucose transporters, glycolytic enzymes, and mitochondrial complexes, thereby supporting critical processes such as proliferation, migration, and differentiation [38]. This metabolic modulation significantly contributes to its pro-fibrotic effects, emphasising the need for an integrated understanding of Src’s influence on cellular functions.

Src modulates glycolysis through multiple mechanisms, including the regulation of key transcription factors like HIF-1α and MYC [43,44,45]. Processes like insulin secretion, phosphorylation-mediated activation of glycolytic enzymes (e.g., hexokinase, PFKFB3, G6PD), and interaction with central metabolic pathways such as the PI3K–AKT–mTOR axis and EGFR signalling are also modulated by Src, highlighting its critical role in energy metabolism [46,47,48,49,50,51].

Several studies have identified a link between Src activity and regulation of the pentose phosphate pathway (PPP) [52,53,54]. In endothelial cells, it is suggested that Src phosphorylation of glucose-6-phosphate dehydrogenase (G6PD) at residues Tyr428 and Tyr507 might modulate G6PD activity [54].

Src has also emerged as a key regulator of mitochondrial function, influencing processes like electron transport chain (ETC) activity, glucose metabolism, and mitophagy [55,56]. Mitochondrial Src (mtSrc) directly phosphorylates ETC complexes and pyruvate dehydrogenase (PDH), affecting mitochondrial respiration and metabolic adaptations [57,58]. However, its role is context dependent, with studies showing both pro- and anti-tumoral effects depending on Src activity levels, cell type, and metabolic plasticity [38,59]. Additionally, Src regulates mitophagy by phosphorylating FUNDC1, inhibiting its activity under stress conditions [56,60,61]. Recent omics studies have highlighted the broad impact of Src on mitochondrial phosphoproteomes and metabolomes, linking it to key metabolic pathways like oxidative phosphorylation, fatty acid metabolism, and the tricarboxylic acid cycle [57,62].

These findings highlight Src’s role as a metabolic regulator with therapeutic potential. While its role in cancer metabolism is well established, Src’s influence on conditions such as fibrosis and glaucoma remains underexplored. Targeting Src in these contexts could disrupt pathological metabolic reprogramming, offering a novel approach for addressing fibrotic diseases and glaucoma-related dysregulation.

## 3. Role of Src in Fibrosis

Fibrosis, characterised by the excessive deposition of extracellular matrix components, is a pathological process that can affect various organs, including the lungs, liver, heart, and kidneys [63,64]. Src has been implicated in the regulation of fibrotic processes, particularly through its role in cellular signalling pathways that control fibroblast activation, proliferation, and migration [63]. Src kinases are activated in response to elevated levels of reactive oxygen species and pro-fibrotic cytokines, such as TGF-β, PDGF, and angiotensin-2, which are commonly elevated in pro-fibrotic disease states [65,66].

LC cells inherently express α-SMA, elastin, COL1A1, and fibronectin, and previous research from our group demonstrated that these cells respond to mechanical stretch, TGF-β1, hypoxia, and oxidative stress by upregulating key ECM genes in a pro-fibrotic manner [67,68,69,70,71,72]. Src kinases modulate TGF-β signalling by phosphorylating and activating the TGF-β type II receptor, as well as the downstream effector c-Abl [73,74].

Recent findings further revealed that increased substrate stiffness alone can induce an activated, myofibroblastic phenotype in previously quiescent, non-glaucomatous LC cells, resembling glaucomatous characteristics [75]. Src promotes the activation of myofibroblasts, which are responsible for excessive collagen production and tissue scarring. The interaction between Src and other signalling molecules, such as integrins and FAK, enhances fibroblast migration and adhesion, facilitating tissue remodelling and fibrosis progression [76]. Through phosphorylation of FAK at multiple sites, Src not only enhances FAK activity but also facilitates the recruitment of adaptor proteins like Grb2 and PI3K, which activate key signaling cascades such as RAS-MAPK and PI3K-AKT [77,78,79].

ECM stiffening, accompanied by both quantitative and qualitative remodelling, is a hallmark of chronic fibrotic diseases, common to numerous systemic and ocular pathologies [80]. We believe that the biomechanical rigidity of the LC, as sensed by its resident cellular population, acts simultaneously as both an initiator and a product of fibrotic remodelling and progressive stiffening in glaucoma [81]. This process appears to operate via a self-perpetuating feedback loop, wherein increasing matrix stiffness further accelerates fibrotic responses and tissue rigidity, thereby exacerbating disease progression.

Through Src’s interactions with FAK, the formation of α-SMA is facilitated [82,83]. In fibroblasts plated on a fibrotic matrix, integrin αV recruits and subsequently activates Src, and this direct interaction between Src and integrin αV is essential for PDGF-BB-induced fibroblast migration [82,83]. Recent research from our group demonstrated that silencing αVβ3 integrin reduced ECM synthesis in LC cells, supporting the interplay between Src and integrins in regulating fibrosis and ECM remodelling, and highlighting αVβ3 integrin as a potential anti-fibrotic target in glaucoma [84]. It has also been shown that αVβ3 integrin signalling in TM cells could have significant impacts on TM function and, ultimately, on glaucoma pathogenesis [85].

Therapeutically, targeting Src in fibrosis is of interest, as preclinical studies have shown that Src inhibitors can reduce fibroblast activation and extracellular matrix deposition in models of lung and liver fibrosis [82,86]. Despite this, clinical development in fibrotic diseases is still in its early stages, and more research is needed to determine the efficacy and safety of Src inhibition in this context.

## 4. Role of Src in Ophthalmology

In the field of ophthalmology, Src plays roles in several processes related to ocular health, including wound healing, angiogenesis, cataract formation, cancer, and intraocular pressure regulation.

Corneal wound healing is partly regulated by Src-mediated signalling pathways [87,88]. Following corneal injury, Src is activated and contributes to the migration and proliferation of corneal epithelial cells, as well as the remodelling of the extracellular matrix, processes that are essential for proper wound closure. Epstein-Barr virus (EBV) infection in human corneal epithelial cells (HCECs) can lead to a mesenchymal fibroblast-like morphology and cause epithelial to mesenchymal transition (EMT) through the activation of PI3K/AKT and ERK by TGF-β1-mediated Syk and Src signalling [89]. Another group found that Src kinase mediates wound-induced EGFR transactivation and participates in a pathway to activate the PI3K-AKT pathway downstream of EGFR in HCECs [90].

Both age-related macular degeneration (AMD) and diabetic retinopathy are characterised by abnormal blood vessel growth, which is driven in part by Src-mediated VEGF signalling [91,92]. While anti-VEGF therapies remain the standard of care, Src inhibitors have also been investigated as potential therapeutic agents, which is discussed further below. In the eye, VEGF acts as a paracrine regulator of conventional outflow facility, secreted by trabecular meshwork cells in response to mechanical stress. This regulation primarily occurs through VEGFR-2 signalling at the Schlemm’s canal (SC) endothelium, a unique structure with a hybrid vascular phenotype that blends blood and lymphatic endothelial cell characteristics [93]. The development of SC, termed “canalogenesis,” involves Prox1 expression and VEGF-C/VEGFR-3 signalling for lymphangiogenesis, alongside VEGFR-2 activity for vessel formation [94]. These processes are essential for SC growth, maintenance, and function. Therapeutically, VEGF-C delivery has been shown to promote SC endothelial proliferation and remodelling, offering a promising strategy for sustained intraocular pressure reduction in glaucoma [95]. The unique vascular biology of SC underscores its critical role in ocular physiology and highlights its potential as a target for innovative glaucoma treatments. However, chronic intravitreal anti-VEGF injections have been found to significantly reduce tonographic outflow facility. Eyes receiving ≥20 injections exhibited a 12% reduction in outflow facility compared to uninjected fellow eyes, with an even greater 46% reduction observed in patients with ocular hypertension [96]. These findings suggest that anti-VEGF therapies may disrupt aqueous humour outflow dynamics, contributing to sustained ocular hypertension and an increased risk of glaucoma development. Thus, understanding and mitigating the effects of VEGF and its signalling pathways on SC function are critical for advancing glaucoma therapies.

This potential for Src family kinase inhibition as a therapeutic approach for retinal vascular diseases, specifically diabetic retinopathy, has been explored elsewhere [97]. Using 12-week-old Akimba mice—a translational model for diabetic retinopathy that mirrors key features of early and advanced disease—researchers conducted an extensive profiling of tyrosine kinase activity in retinal tissues. Their findings revealed an increase in tyrosine kinase activity, with a particular emphasis on Src-FAK family kinases, a signalling hub confirmed to be hyperactive via Western blot analysis. The team further examined the effects of Src and FAK family kinase inhibitors in angiogenesis assays, both in vitro (using HUVEC tube formation) and ex vivo (organotypic choroidal sprouting). Notably, a novel selective Src inhibitor, eCF506, showed significant suppression of angiogenesis, performing comparably to dasatinib, a broader tyrosine kinase inhibitor, without causing toxicity in endothelial cells [97]. This research not only highlights Src family kinases as central players in the hyperactive tyrosine kinome of the diabetic retina but also suggests that targeting Src kinases could be an effective therapeutic strategy to control angiogenesis in retinal diseases. For other ocular conditions involving pathological neovascularisation or ECM remodelling, such as age-related macular degeneration or glaucoma, Src inhibition might offer a similarly promising approach, particularly given its role in cellular signalling and tissue remodelling.

Another study identified the VEGF/VEGFR2/Src signalling pathway as a key driver in the breakdown of the inner blood–retinal barrier (iBRB) under diabetic conditions, primarily through early phosphorylation and subsequent downregulation of vascular endothelial (VE)-cadherin [98]. The disruption of VE-cadherin compromises endothelial barrier integrity, exacerbating diabetic retinopathy. Notably, erythropoietin (EPO) administration was shown to preserve VE-cadherin expression and barrier function by inhibiting VEGF/VEGFR2/Src signalling. These findings suggested that targeting Src in this pathway could provide therapeutic benefits in preserving vascular integrity, offering insights relevant to addressing fibrosis and barrier dysfunction in glaucoma.

In lens epithelial cells, serum-induced Src kinase activation promotes cell migration, disrupts cell–cell junctions, and drives the cells toward a mesenchymal phenotype [99]. This Src-mediated EMT highlights Src’s role in cellular plasticity. Another study demonstrated that Src kinase, when activated by inflammatory factors and high glucose, promoted EMT in lens epithelial cells, enhancing proliferation, migration, and fibrotic potential [100]. Conversely, silencing Src reduced these effects, underscoring Src’s critical role as a driver of EMT and fibrosis in lens pathology. Another study highlighted the role of SFK activation in maintaining Na,K-ATPase activity in the lens, which is critical in preventing cataract formation [101]. Cataracts, often associated with age-related vision loss, arise due to disruptions in Na,K-ATPase-mediated ion transport, leading to imbalanced sodium and potassium levels [102]. Calcium influx through TRPV4 channels was found to activate SFK, leading to increased cAMP production and subsequently enhancing Na,K-ATPase activity [101]. The role of TRPV4 extends to glaucoma pathology, particularly in the TM. Chronic mechanical stress, as seen in elevated IOP, promotes cytoskeletal remodelling in TM cells through actin polymerisation, focal adhesion complex formation, and Rho-associated protein kinase (ROCK) activation [103]. TRPV4 channels mediate calcium influx in response to membrane stretch, which, in conjunction with ROCK signalling, drives the assembly of focal adhesions and cytoskeletal remodelling. Pharmacological inhibition of TRPV4 or ROCK suppresses these processes, demonstrating their critical roles in the mechanical tuning and stiffness of TM cells [103]. Chronic activation of these pathways under sustained mechanical stress may contribute to pathological tissue remodelling, impaired aqueous outflow, and glaucoma progression. These findings underscore the interconnected roles of Src and TRPV4 signalling in regulating ocular tissue mechanics, providing insights for therapeutic strategies targeting fibrosis and elevated IOP in glaucoma.

As outlined above, research has identified Src as a crucial activator of the ERK1/2 pathway in primary uveal melanoma (UM), indicating potential for Src inhibition as a treatment strategy in primary UM [26]. However, as previously stated, metastatic UM cells showed reduced dependency on Src and ERK1/2 signalling, suggesting that Src inhibition may have limited efficacy in advanced stages, where other proliferative pathways predominate.

## 5. Role of Src in Glaucoma

### 5.1. Src in TM and Aqueous Humour Dynamics

One of the key features of glaucoma is increased resistance to aqueous humour outflow, leading to elevated IOP [104]. Src plays a role in the regulation of aqueous humour dynamics, particularly in the trabecular meshwork, which is responsible for fluid drainage from the anterior chamber of the eye [105]. Activation of Src in the trabecular meshwork has been shown to affect cell contractility and extracellular matrix turnover, both of which influence outflow facility and IOP [7].

The effects of Src inhibitors on IOP and TM cells in ocular normotensive rabbits has also been studied [7]. The Src inhibitors PP2, PP1, and damnacanthal were found to significantly lower IOP following intracameral injection. Among the evaluated Src inhibitors, PP2 displayed the strongest efficacy in lowering IOP. The study also found that PP2 decreased the trans-epithelial electrical resistance (TEER) of TM cell layers in a dose-dependent manner ranging from 0.1 mM to 100 mM. The maximal efficacy of PP2 on TEER was a reduction to 71.7% relative to the vehicle-treated group at 100 mM. PP2 decreased the adhesion of TM cells to culture surfaces either uncoated with specific ECM proteins dose dependently or coated with extracellular matrix proteins, such as laminin I, fibronectin, and collagen type I, and basement membrane extraction. Tyrosine phosphorylation of focal adhesion kinase and p130Cas was decreased by PP2. On the other hand, major changes in the actin staining of TM cells were not able to be detected after PP2 treatment, although quantitative analysis showed that PP2 induced some morphological changes, which were in a different direction to those caused by Y-27632, a Rock inhibitor. The study suggested that the efficacy of Src inhibitors in lowering IOP may be partially attributed to their effects on TM cell adhesion and integrity, indicating that SFK inhibitors may act on TM cells in a manner that is distinct from Rock inhibitors.

### 5.2. Src and Cyctoskeletal Dynamics in Glaucoma

Dasatinib, an inhibitor of Src and Abl kinases, was shown to disrupt key cytoskeletal and cytoskeletal-associated (cytoskeletome) proteins in dexamethasone-treated human trabecular meshwork (HTM) cells, with potential implications for IOP regulation in glaucoma [106]. Using label-free mass spectrometric quantification, the researchers identified elevated levels of proteins involved in actin stress fibre formation, contraction, actin network crosslinking, cell adhesion, and Wnt signalling, including LIMCH1, ArgBP2, CNN3, ITGBL1, CTGF, palladin, FAT1, DIAPH2, EPHA4, SIPA1L1, and GPC4. Many of these proteins colocalised with the actin cytoskeleton and exhibited altered distribution patterns when TM cells were treated with dasatinib in combination with dexamethasone. Specifically, dasatinib disrupted the cytoskeletal association of palladin and CNN3, suggesting that Src and Abl kinase activity is crucial for the tyrosine phosphorylation-dependent interaction between these proteins and the actin cytoskeleton. These findings underscore the therapeutic potential of Src inhibition in modulating cytoskeletal pathways linked to fibrosis and IOP regulation in glaucoma.

### 5.3. Src in RGC Survival and Neuroprotection

Src has been suggested as a mechanistic link between inflammation and cancer [107]. The precise mechanisms underlying the injury and eventual degeneration of retinal ganglion cell (RGC) axons remain poorly understood; however, preliminary evidence indicates that neuroinflammatory responses initiated by astrocytes, microglia, and other immune cells derived from the bloodstream occur within the optic nerve head (ONH), implying that inflammation may play a central role in the pathogenesis of glaucoma [108,109,110]. Inhibition of Src has been shown to alleviate microgliosis and reduce inflammatory factor levels in Parkinson’s disease mouse models, suggesting its role in modulating neuroinflammation [111]. Additionally, Src inhibition mitigated dopaminergic neuron loss and improved motor function, highlighting its potential as a therapeutic target. These findings underscore the importance of Src in neuroinflammatory processes and propose Src inhibition as a promising strategy for treating glaucoma and other neuroinflammation-driven diseases. Furthermore, Src has been associated with neurodegenerative processes in glaucoma. Src activation has been linked to RGC apoptosis, which is a critical event in glaucoma-related optic nerve damage [10]. The authors found that Src homology region 2-containing protein tyrosine phosphatase 2 (Shp2) underwent activation in RGCs in animal models of glaucoma as well as in human glaucoma tissues. Overexpression of Shp2 in RGCs promoted endoplasmic reticulum (ER) stress and apoptosis, along with functional and structural deficits in the inner retina. The overexpression of Shp2 increased ER stress markers such as CHOP and phospho-PERK in the inner retina, while Shp2 silencing suppressed this ER stress response in the glaucoma model. ER stress has been implicated in RGC degeneration in glaucoma [112]. This proves that Shp2 is a critical regulator of RGC survival in experimental glaucoma. Targeting Src may therefore represent a therapeutic strategy for neuroprotection in glaucoma, in addition to its potential role in controlling IOP.

Dock3, a protein expressed in the central nervous system, exerts significant neuroprotective effects in the retina and optic nerve, largely through mechanisms independent of its traditional guanine exchange factor (GEF) activity [113]. A key aspect of Dock3’s action involves inhibiting the SFK member Fyn, which stabilises the N-methyl-D-aspartate (NMDA) receptor on the cell surface [114]. By blocking Fyn, Dock3 promotes NMDA receptor degradation, reducing excitotoxicity and oxidative stress, thereby enhancing RGC survival [115]. Additionally, Dock3 has been shown to mitigate oxidative damage through suppression of the apoptosis signal-regulating kinase 1 (ASK1) pathway, which is activated in response to cellular stress and is implicated in RGC loss in glaucoma models [116,117]. These insights suggest that targeting Src signalling could provide neuroprotection in glaucoma, offering a strategy to counteract fibrosis-related damage, excitotoxicity, and oxidative stress, with Dock3 signalling as a promising therapeutic target.

In a chronic ocular hypertension (COH) rat model, elevated EphB/ephrinB signalling was associated with RGC apoptosis [118]. EphB receptors and ephrinB ligands are a subset of the RTK family involved in bidirectional signalling that regulates cell–cell interactions. EphrinBs are transmembrane proteins, and their interaction with EphB receptors mediates cytoskeletal dynamics, cell migration, and morphological changes, playing key roles in development and cellular organisation [119]. Both EphB1 and ephrinB2 levels were significantly higher in COH compared to controls, with EphB1 and ephrinB2 expressed in Müller cells and ephrinB2 also present in RGCs, establishing an EphB1/ephrinB2 reverse signalling pathway in RGCs [118]. This signalling pathway’s activation led to increased ephrinB phosphorylation, which contributed to RGC apoptosis through mechanisms involving the Src family kinase (SFK) pathway. Specifically, the SFK inhibitor PP2 blocked EphB/ephrinB-induced RGC apoptosis by preventing the phosphorylation and subsequent endocytosis of GluA2-containing α-amino-3-hydroxyl-5-methyl-4-isoxazole-propionate (AMPA) receptors, which is crucial for RGC survival. Further studies confirmed that PP2 reduced GluA2 trafficking and RGC apoptosis, indicating that Src inhibition could be a potential therapeutic approach for neuroprotection in glaucoma. The group additionally showed that ephrinA3/EphA4 forward signalling similarly promoted GluA2 endocytosis and RGC apoptosis in COH models, further supporting the role of Eph/ephrin signalling and Src kinases in glaucomatous degeneration [120]. Collectively, these findings suggest that Src inhibition could serve as a viable strategy for preventing RGC loss in glaucoma by modulating Eph/ephrin-mediated signalling and AMPA receptor trafficking.

### 5.4. Src and TGF-β-Mediated Fibrosis in Glaucoma

It has been previously shown that Src activity plays a crucial role in TGF-β-induced IOP elevation, and that Src represents a potential therapeutic target for the treatment of glaucoma [8]. Injection of the Src kinase inhibitor dasatinib suppressed the IOP elevation caused by TGF-β2 in rat eyes. In human trabecular meshwork cells, TGF-β2 treatment activated Src signalling and induced cytoskeletal remodelling, cell adhesion, and ECM accumulation. Src was activated via TGF-β2-induced upregulation of the Src scaffolding protein CasL, which mediated the assembly of focal adhesions, cytoskeletal remodelling, and ECM deposition. Activation of Src suppressed the expression of tissue plasminogen activator (tPA), thereby attenuating ECM degradation. Dasatinib ameliorated the TGF-β2-induced changes in the contractile and adhesive characteristics of trabecular meshwork cells, as well as ECM deposition [8].

Another study evaluated the effects of Src inhibitors, such as bosutinib, dasatinib, and SU-6656, on scleral myofibroblast differentiation, with dasatinib demonstrating significant inhibition [121]. They identified seven inhibitors as showing over 80% reduction in ECM binding during the initial screen. Subsequently, three kinase inhibitors, rottlerin, PP2 (4-amino-3-(4-chlorophenyl)-1-(t-butyl)-1H-pyrazolo [3,4-d] pyrimidine), and tyrphostin 9, were verified to reduce TGF-β-induced αSMA expression and cellular contractility. The study further evaluated the effects of three Src inhibitors, where dasatinib was found to significantly inhibit TGF-β-induced ECM synthesis, αSMA expression, and cellular contractility at nanomolar dosages. The researchers also found that subconjunctival injection of dasatinib reduced intraocular pressure-induced scleral fibroblast proliferation [121].

### 5.5. Src and YAP/TAZ Pathways in Glaucoma

Specific molecular players like Src family kinases, as well as microRNA-137 (miR-137) and YAP, have been investigated for their roles in regulating cellular function, including cellular growth, apoptosis, ECM protein expression, and the induction of RGC apoptosis [9,118,122].

It has been shown that miR-137 was downregulated in H_2_O_2_-induced HTM cells, and overexpression of miR-137 attenuated H_2_O_2_-induced cell growth inhibition, apoptosis, and elevated ECM protein expression [122]. The study found that miR-137 inhibited the activation of the YAP/TAZ pathway by directly targeting Src. Overexpression of Src or activation of the YAP/TAZ pathway partly reversed the effects of miR-137 on cell viability, apoptosis, and ECM protein expression. The study’s findings highlight the significance of miR-137 in regulating cell proliferation, apoptosis, and ECM protein expression in glaucoma. Inhibiting Src might upregulate miR-137 and reduce apoptosis, elevated ECM expression, and cell growth inhibition.

The transcriptional activity of YAP, a key regulator in cell growth and survival, is tightly controlled by its phosphorylation status, which determines its cellular localisation. Phosphorylation at serine 127 (pYAP[s127]) by the Hippo pathway promotes YAP’s cytoplasmic retention and limits its activity, whereas phosphorylation at tyrosine 357 (pYAP[y357]) by SFKs enhances its nuclear accumulation and upregulates gene transcription through TEAD binding [123,124,125,126]. Notably, Src-mediated phosphorylation at tyrosine 357 can drive YAP nuclear translocation independently of serine 127 status and simultaneously inhibit the Hippo pathway, effectively enhancing YAP’s transcriptional impact [127]. Nuclear accumulation and upregulation of YAP are key contributors to the pathogenesis of various cancers and multisystem fibrotic diseases [128]. In vitro, cancer-associated fibroblasts exposed to a stiffened microenvironment exhibit YAP nuclear translocation, enhanced transcription of profibrotic target genes, and increased proliferation, highlighting its role in mechanotransduction and fibrosis [129]. Our group previously showed that glaucomatous lamina cribrosa cells exhibited increased YAP expression, nuclear localisation, and proliferation compared to non-glaucomatous controls, with these effects amplified on stiff substrates mimicking glaucomatous conditions [9]. YAP activation was associated with upregulation of myofibroblastic markers such as α-SMA and Col1A1, while its inhibition with verteporfin reduced YAP-mediated cellular activation and proliferation despite the stiffened microenvironment. Increased ECM stiffness and elevated TGF-β2 levels in POAG drive enhanced YAP/TAZ nuclear localisation in HTM cells via focal adhesion modulation and cytoskeletal rearrangement [130]. These effects are mediated by ERK and ROCK pathways and are reversed by F-actin depolymerisation or YAP/TAZ inhibition with verteporfin. YAP/TAZ activation contributes to HTM dysfunction by promoting ECM remodelling, focal adhesion formation, and contractility, suggesting a critical pathological role in glaucoma progression. These insights collectively indicate that Src inhibition could serve as a therapeutic strategy to prevent YAP-driven fibrotic responses and potentially modulate IOP in glaucoma by limiting YAP’s transcriptional activation in fibrotic pathways.

## 6. Conclusions

The Src proto-oncogene plays a pivotal role in diverse cellular processes, with far-reaching implications for human health and disease. Beyond its well-characterised role in promoting tumour growth and metastasis in cancer, Src regulates fibroblast activation and extracellular matrix deposition in fibrosis, underscoring its importance in pathological tissue remodelling. Emerging evidence highlights its influence on cellular metabolism, including glycolysis, the pentose phosphate pathway, and mitochondrial function, which may underpin its role in driving disease processes.

In ophthalmology, Src is implicated in critical pathways governing wound healing, angiogenesis, cataract formation, cancer progression, aqueous humour outflow, and neurodegeneration. These processes link Src to various ocular diseases, including corneal injury, cataract, age-related macular degeneration, diabetic retinopathy, uveal melanoma, and glaucoma. In glaucoma specifically, Src’s involvement in regulating trabecular meshwork function, fibrosis, RGC death, and intraocular pressure suggests that it may be a key therapeutic target. However, the complexity of Src signalling networks poses challenges for drug development, and further research is needed to elucidate the precise role of Src in glaucoma pathogenesis and validate its potential as a therapeutic target.

## Data Availability

Not applicable.
